# Delta albumin is a better prognostic marker for complications following laparoscopic intestinal resection for Crohn’s disease than albumin alone – A retrospective cohort study

**DOI:** 10.1371/journal.pone.0206911

**Published:** 2018-11-13

**Authors:** Catharina Müller, Anton Stift, Stanislaus Argeny, Michael Bergmann, Michael Gnant, Sebastian Marolt, Lukas Unger, Stefan Riss

**Affiliations:** Department of Surgery, Division of General Surgery and Comprehensive Center for Perioperative Medicine, Medical University of Vienna, Vienna, Austria; University of South Alabama Mitchell Cancer Institute, UNITED STATES

## Abstract

**Purpose:**

Little is known about the perioperative dynamic of albumin and its effect on surgical outcome in Crohn’s disease. Thus, we aimed to assess postoperative changes of albumin levels and their potentially predictive role for complications after laparoscopic intestinal resections.

**Methods:**

We identified 182 patients who underwent laparoscopic intestinal resection for symptomatic Crohn´s disease between 2000 and 2014 for this retrospective cohort study. Pre- and postoperative serum albumin levels (within 4 days) were recorded retrospectively and proportional postoperative reduction (delta (Δ) albumin) was calculated. Complications were defined according to the Clavien-Dindo classification. Univariate and multivariate analysis describing an eventful postoperative course were conducted.

**Results:**

Complications were found in 22.5% (n = 41), 6% (n = 11) developed major complications defined as Clavien Dindo III-V and 16.5% (n = 30) had minor complications (Clavien Dindo I-II). The median Δ albumin was 22.75% (range: -18.46–47.14%). Delta albumin was found to be significantly higher in patients who developed complications after surgery (p = 0.03). Notably, neither preoperative (p = 0.28) nor postoperative albumin levels (p = 0.41) taken as absolute numerical values correlated with an eventful course following intestinal resection. In the multivariate analysis, based on a cut-off of 24.27%, Δ albumin remained an independent factor for surgical complications (p = 0.04, OR 2.232) next to conversion rate (p<0.001, OR 5.577) and the presence of an inflammatory mass (p = 0.003, OR 0.280).

**Conclusion:**

Δ albumin is a better prognostic marker for an eventful postoperative course after laparoscopic surgery in patients with Crohn’s disease in comparison to albumin alone.

## Introduction

The majority of patients suffering from Crohn’s disease (CD) require surgery once in their lifetime [1]. Notably, patients with an eventful postoperative course tend to have an impaired long-term outcome reflected by a higher rate of recurrence and re-operation [2, 3]. Thus, a reliable marker to predict a complicated course following surgery would be helpful to guide clinical management and to identify patients at risk for complications. Additionally, in the era of Enhanced Recovery After Surgery–ERAS–protocols, with proven beneficial outcome but also attempted short hospital stay [4], a marker for safe discharge would be helpful in daily clinical practice. Various studies have focused on risk factors for postoperative complications in CD. Amongst others, anemia, the use of glucocorticosteroids or biologicals in the perioperative period and the presence of intra-abdominal infection have been associated with increased risk for complications [5]. However, a reliable marker to predict short-term outcome is still lacking.

Serum albumin is known as a negative acute phase protein and a marker for protein-energy malnutrition (PEM). In patients with CD hypoalbuminemia is a frequent finding due to systemic inflammation, poor nutritional state and gastrointestinal protein loss [6, 7]. Notably, data about low preoperative albumin levels as a risk factor for surgical complications in patients with CD remain controversial. Postoperative albumin levels were associated with complications after various surgical procedures [8, 9] but data for patients with CD are scarce [10]. Furthermore, the comparison of available literature remains difficult because cut-off values defining low albumin levels varied immensely among studies.

Recent studies revealed that the decrease of albumin following surgery was associated with extent of surgical trauma and consequently with an elevated number of adverse events [11]. Whether this is true for laparoscopic surgery in CD with potentially low surgical stress induction and less tissue trauma is unknown. Thus, our large cohort study was designed to evaluate perioperative serum albumin dynamics in regard to complication rate in patients undergoing laparoscopic surgery for symptomatic CD. We hypothesize that the decrease of serum albumin levels in the early postoperative period (day 1 to day 4) represented as Δ albumin is related to postoperative morbidity.

## Materials and methods

### Study design and data collection

We retrospectively identified 232 patients who underwent laparoscopic intestinal resection for symptomatic CD at a tertiary referral center between 2000 and 2014. Fifty patients had to be excluded due to unavailable laboratory data, leaving 182 patients (106 males, 76 females) for final analysis.

All operations were conducted by a single colorectal team specialized in treating CD patients. The technique of laparoscopic surgery has been previously described in detail. Conversion was defined as extension of planned incision [12]. Perioperative management was similar for all patients with respect to prophylactic antibiotics, bowel preparation and fluid management. Postoperative management was standardized. Patients received liquids (tea, soup, yoghurt) from the fist postoperative day and light solid diet was introduction the third postoperative day. In the first days 2000ml parenteral fluids were administered according to individual liquid intake.

Pre- and postoperative serum albumin levels (within 4 days) were recorded and proportional postoperative reduction (delta (Δ) albumin) was calculated by following formula: (preoperative albumin–postoperative albumin)/preoperative albumin x 100 (%). Hypoalbuminemia was defined as albumin level < 35g/l.

All data were obtained from our institutional database and individual chart review retrospectively. Basic characteristics consisted of age at the time of surgical intervention, gender, body mass index (BMI), smoking history, immunosuppressive therapy and extent of disease (abscess formation, fistulation, stenosis, inflammatory mass). Operative data included urgency of operation, operation time and extend of surgery (‘complex surgery’ was defined by the need of more than one resection and/or additional stricture plastic).

Postoperative complications within 30 days after operation were recorded and classified according to Clavien Dindo classification [13]. Additionally, complications were divided into major (Clavien Dindo III-V) and minor complications (Clavien Dindo I-II).

The study was approved by the Medical University of Vienna’s institutional ethical review board (ECS 2156/2017). The study has been structured according to STROCSS statement [14] and was approved by the institutional ethical review board.

### Statistical analysis

IBM Statistical Package for the Social Sciences (SPSS) Version 24 for Mac (SPSS Inc., Chicago, IL, USA) was used for statistical analysis. Student’s t-test was performed to calculate group differences for continuous parameters if normally distributed and χ^2^-test for categorical variables. Otherwise, Mann-Whitney-U-Tests and fisher’s exact test were used accordingly. A significance level of two sided p-value ≤ 0.05 was considered to denote statistical significance. Prognostic value was calculated by the receiver operating characteristics (ROC) curve and expressed as area under the curve with an asymptomatic 95% confidence interval (CI). We calculated the Youden-Index (the sum of sensitivity and specificity) to find the best predictive value and to define a cut-off for group classification. Furthermore, uni- and multivariate analysis was conducted with linear regression models. First univariate analysis was performed for all baseline characteristics. In a second step significant parameters with a p-value of < 0.05 were included in a multivariate regression analysis with step-wise forward selection.

## Results

### Demographic data

Detailed patient’s characteristics are listed in Table 1.

**Table 1:** Baseline characteristics.

	All (median range)	Δalbumin^high^	Δalbumin^low^	p value
**Patients characteristics**				
Age (y)	31.57 (15.42–76.47)	35.72 (15.42–66.05)	32.41 (16.38–76.47)	p = 0.78
Gender				
male	n = 106 (58.2%)	n = 44 (55%)	n = 62 (60.8%)	p = 0.45
female	n = 76 (41.8%)	n = 36 (45%)	n = 40 (39.2)	
BMI (kg/m^2^)	21.37 (12.22–40.61)	21.86 (12.22–40.61)	21.81 (14.53–34.15)	p = 0.94
Smoking				
yes	n = 89 (47.3%)	n = 38 (47.5%)	n = 52 (51%)	p = 0.76
no	n = 90 (49.5%)	n = 39 (48.8%)	n = 47 (46.1%)	
Immunosuppression				
yes	n = 100 (54.9%)	n = 45 (56.3%)	n = 55 (53.9)	p = 0.73
no	n = 48 (26.4%)	n = 20 (25%)	n = 28 (27.5%)	
Number of prior operations for CD				
0	n = 146 (80.2)	n = 66 (82.5%)	n = 80 (78.4%)	p = 0.85
1	n = 25 (13.7%)	n = 9 (11.3%)	n = 16 (15.7%)	
2	n = 9 (4.9%)	n = 4 (5%)	n = 5 (4.9%)	
3	n = 2 (1.1%)	n = 1 (1.3%)	n = 1 (1.3%)	
**Surgical characteristics**				
Conversion				
yes	n = 36 (19.8%)	n = 21 (26.3%)	n = 15 (14.7%)	p = 0.06
no	n = 146 (80.2)	n = 59 (73.8%)	n = 87 (85.3%)	
Urgency of operation				
elective	n = 173 (95.1%)	n = 77 (96.3%)	n = 96 (94.1%)	p = 0.73
acute	n = 9 (4.9%)	n = 3 (3.8%)	n = 6 (5.6%)	
Operation time (min)	135 (50–370)	158 (70–360)	145 (50–370)	p = 0.18
Complexity				
simple	n = 125 (68.7%)	n = 55 (68.8%)	n = 70 (68.6%)	p = 1.00
complex	n = 57 (31.3%)	n = 25 (31.3%)	n = 32 (31.4%)	
**Disease characteristics**				
Stenosis				
no	n = 46 (25.3%)	n = 24 (30%)	n = 22 (21.6%)	p = 0.18
1	n = 116 (63.7%)	n = 45 (56.3%)	n = 71 (69.6)	
>1	n = 20 (11%)	n = 11 (13.8%)	n = 9 (8.8%)	
Abscess				
no	n = 141 (77.5%)	n = 63 (78.8%)	n = 78 (76.5%)	p = 0.73
yes	n = 41 (22.5%)	n = 17 (21.3%)	n = 24 (23.5%)	
Fistula				
no	n = 80 (44%)	n = 34 (42.5%)	n = 46 (45.1%)	p = 0.95
1	n = 77 (42.3%)	n = 35 (43.8%)	n = 42 (41.2%)	
>1	n = 25 (13.7%)	n = 11 (13.8%)	n = 14 (13.7%)	
Inflammatory mass				
no	n = 98 (53.8%)	n = 42 (52.5%)	n = 56 (54.9%)	p = 0.77
yes	n = 84 (46.2%)	n = 38 (47.5%)	n = 46 (45.1%)	

Detailed baseline characteristics of patients operated on laparoscopically for CD.

At the time of surgery 100 patients (55%) received immunosuppressive therapy of whom 70 patients received glucocorticoids, 14 patients biologicals and 30 patients azathioprin.

In 123 (67.6%) patients an ileocoecal resection was performed, followed by colonic resections (n = 46, 25.2%). Ten patients (5.5%) underwent segmental small bowel resection and three patients (1.6%) required rectal resection. One hundred and twenty-five (68.7%) of these operations qualified for simple and 57 (31.3%) for complex surgeries as defined above.

A conversion rate of 19.8% (n = 36) was observed. Conversion was performed due to bleeding (n = 1), adhesions (n = 12), anatomical difficulties (n = 3), massive inflammation (n = 4), fistula (n = 4), perforation (n = 1) or others (n = 2).

The total rate of postoperative complications, defined as Clavien Dindo Score > 0, was 22.5% (n = 41). Eleven (6%) patients developed major complications (Clavien Dindo III-V) and 30 patients (16.5%) had minor complications (Clavien Dindo I-II). No patient died in the postoperative period.

Postoperative paralytic ileus defined as gastric tube re-insertion or presence of corresponding symptoms [15] was the most frequent minor complication accounting for 34.1% of all complications (n = 14). Major complications necessitating surgical revision or radiologic intervention consisted of two patient with mechanical bowel obstruction (1.1%) and 6 patients with anastomotic leakage (3.3%). In addition, one patient (0.6%) each needed reoperation because of drain dislocation, postoperative bleeding and intra-abdominal infection. Detailed complication rates are listed in Table 2.

**Table 2:** Postoperative complications.

Clavien Dindo classification	n/% of total population
Grade I	n = 6/3.3
Grade II	n = 24/13.2
Grade III und IIIb	n = 10/5.5
Grade IV und V	n = 1/0.5

Complications defined according to the Clavien Dindo classification.

### Serum albumin dynamics and complications

Forty-nine patients (26.9%) presented with hypoalbuminemia before surgery and 170 patients (93.4%) developed hypoalbuminemia postoperatively. Low preoperative albumin levels significantly correlated with lower BMI (p = 0.001). Prior surgery the median albumin level was 38.5g/l (range: 16.80–50.10), which declined to a median level of 29.05g/l (range: 14.50–40.60) postoperatively. Neither preoperative (p = 0.28) nor postoperative albumin levels (p = 0.40) were significantly associated with postoperative morbidity.

We observed a substantial proportional decrease of albumin, expressed as Δ albumin, with a median Δ albumin of 22.75% (range: -18.46–47.14%), which corresponded to the median absolute albumin decrease of 8.75 g/l (range: -4.80–20.60). Notably, Δ albumin was significantly higher in patients developing complications (p = 0.03). The median Δ albumin showed 22.3% (range: -18.46–47.14%) for patients with an uneventful course in contrast to 25.75% (range: 7.73–41.19%) for patients with eventful course following surgery.

Additionally, Δ albumin increased in relation to the severity of complications as median Δ albumin rose from 22% without complication to 25% for minor to 31% for major complications (p = 0.01).

### Predictive value of Δ albumin for postoperative complications

**Fig 1:**
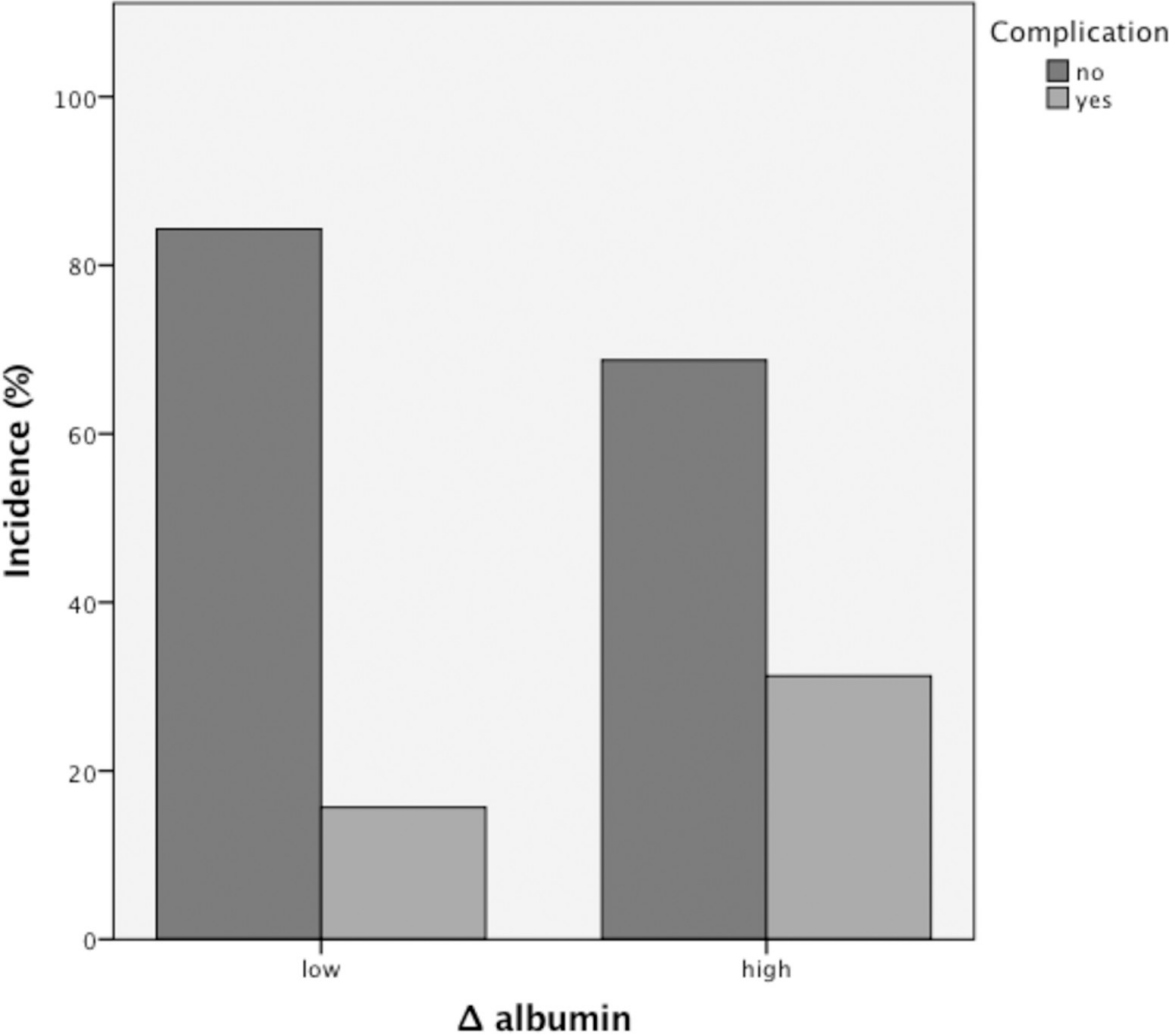
Perioperative albumin decline and the incidence of complications. Patients with a high perioperative albumin decline of more than 24.27% are at greater risk to developed postoperative complications (31.3% vs. 15.7%).

As we found significant differences of Δ albumin levels in patients with an eventful course following surgery (Fig 1) we used the ROC analyses to evaluate its predictive value. The predictive value expressed as an area under the curve (AUC) was 0.612 (p = 0.03, AUC 61.2%, 95% CI 0.514–0.710) with a cut-off in the ROC analyses of 24.27% calculated with the Youden index (1.22, sensitivity and specificity of 61%).

### Univariate analysis

We used the maximal threshold calculated in the Youden index for group classification and accordingly divided our population in Δ albumin^high^ (> 24.27%) and Δ albumin^low^ (< 24.27%). The two cohorts, Δ albumin^high^ and Δ albumin^low^ were comparable for all characteristics as seen in Table 1. Univariate analysis showed a significant correlation between Δ albumin (p = 0.014), conversion (p<0.001), age at the time of surgery (p = 0.01, median 30y vs. 38y), operation time (p = 0.01, median 175min vs. 130min) and the presence of an inflammatory mass (p = 0.006) and surgical morbidity (Table 3).

**Table 3:** Univariate analysis for complications according to basic characteristics and multivariate analysis with stepwise forward regression.

	Univariate Regression for occurrence of complications			Multivariate Regression for occurrence of complications		
Characteristics	p	OR	95% CI	P	OR	95% CI
Albumin preoperative	0.28	1.040	0.969–1.115			
Albumin postoperative	0.40	0.967	0.895–1.046			
Δ albumin^low^/ Δ albumin^high^	0.01*	1.443	1.198–4.984	0.04*	2.232	1.029–4.844
Conversion	<0.001*	5.348	2.426–11.791	<0.001*	5.577	2.370–13.121
Urgency	0.12	0.340	0.87–1.330			
Complexity	0.75	0.883	0.413–1.888			
Operation time	0.01*	1.007	1.002–1.013			
Age	0.02*	1.034	1.006–1.062			
Gender	0.45	1.322	0.645–2.709			
BMI	0.16	1.060	0.977–1.149			
Smoking	0.48	1.285	0.637–2.588			
Immuno- suppression	0.33	1.523	0.650–3.567			
Number of prior surgeries (Crohn related)	0.56	1.175	0.680–2.030			
Stenosis	0.97	0.987	0.545–1.788			
Abscess	0.08	0.405	0.148–1.111			
Fistula	0.92	1.026	0.623–1.687			
Inflammatory mass	0.006*	0.342	0.159–0.735	0.003*	0.280	0.121–0.648

* p<0.05, significant

### Multivariate regression analysis

For multivariate regression analysis we used stepwise forward exclusion. The results are shown in Table 3. Delta albumin at a cut-off of 24.27% remained an independent risk factor for complications next to conversion and the presence of an inflammatory mass. Twenty-five patients (31.3%) with a great decline of albumin in contrast to 15.7% of patients with low Δ albumin had an eventful postoperative course (p = 0.02). Fifty percent (n = 18 out of 36) with extension of incision suffered from complications while only 15.8% (n = 23 out of 146) of operations completed laparoscopically were followed by an eventful course (p<0.001). Surprisingly, patients with an inflammatory mass had a lower rate of postoperative complications (13%) in contrast to 30.6% without inflammatory mass (p = 0.005).

## Discussion

In this cohort study we could demonstrate that the perioperative albumin decline represented a significant risk factor for adverse outcome following laparoscopic intestinal resection for CD. Neither pre- nor postoperative albumin levels correlated with an eventful course after surgery, pointing out the value of perioperative albumin dynamics.

The impact of hypoalbuminemia on surgical outcome has been evaluated in various studies. Notably, interpretation of literature is insidious for patients with CD due to inconsistency of albumin cut-offs used to define high and low levels of albumin. Morar et al. found hypoalbuminenia to be a risk factor for intra-abdominal septic complications after ileocolonic resection using a cut-off of 25g/l [5] while others observed no correlation with surgical outcome after intestinal anastomosis performed during surgery for CD using a threshold of 40g/l [3]. In general, a trend towards increased complications in patients with a low albumin level was found in several studies [16, 17], while recently one prospective study observed a higher risk for surgical site infections but not for the overall complication rate [18]. In line with this finding, we cannot confirm preoperative serum albumin level alone to be a risk factor for an eventful course following surgery.

Preoperative albumin may be interpreted as a marker for long-term nutritional condition. In our study 26.9% of patients presented with hypoalbuminemia prior surgery, which was significantly related to BMI. However, it is widely known that many factors affect serum albumin levels such as hepatic function, inflammatory response, loss of albumin or hyper-hydration. Thus, it is proposed that albumin levels rather reflect severity of illness than nutritional state alone [19] and low albumin levels have been shown to predict activity of CD [20].

Different medical conditions influence albumin synthesis, catabolism and distribution. On a translational level a decreased gene transcription with consecutive reduction of mRNA and albumin synthesis was found in inflammation mainly caused by IL-6 and TNF-α while transcription of mRNA for acute-phase proteins increased [21]. A pilot study recently showed no difference in albumin synthesis rates postoperatively even though serum albumin levels reduced significantly (about 34%) [22]. Therefore, albumin decrease after surgery may be caused by other factors such as capillary leak, intraoperative blood loss, dilution due to hyper-hydration or to a little extend catabolism rather than altered albumin metabolism [23, 24]. Notably, decline of albumin levels occurs within hours after surgery and remains stable for various days postoperatively [25, 26]. As a consequence, albumin may be a fast and robust marker for systemic inflammatory response following surgery, which, in contrast to many other specific inflammatory factors, is easily available in daily clinical practice.

Recently, a prospective study showed that the decline of albumin reflected the extend of surgery and correlated with surgical trauma and postoperative morbidity in patients after major abdominal surgery [11]. Similarly, we found a significantly higher decline of albumin in patients with an eventful course and revealed a clear association with severity of complications.

Laparoscopic surgery causes less tissue trauma and induces less systemic inflammatory response in comparison to open surgery [27]. Thus, the laparoscopic approach was used as a surrogate parameter for extend of surgery when Δ albumin and surgical stress was evaluated [11]. To our knowledge this is the first study to date that aimed to investigate the correlation between the decline of albumin and surgical morbidity particularly in laparoscopic surgery for CD. Interestingly, we found a high overall postoperative decrease of albumin in our population with a median Δ albumin of 22.75% despite using only a minimal invasive approach with consequently less surgical stress.

The predictive role of Δ albumin for surgical outcome has been investigated by Ge et al. who found that a decrease of serum albumin of 15% within the first two days after surgery represented an independent risk factor for morbidity in patients undergoing elective colectomy or rectal resection for inflammatory bowel disease (IBD) [28]. Another study of patients undergoing rectal resection for rectal cancer observed an association of high Δ albumin with an eventful postoperative course [29].

Our data do not allow Δ albumin to be used as a diagnostic marker for its sensitivity and specificity of only 61% but we may define risk groups at a certain Δ albumin cut-off. Delta albumin at a cut-off of 24.27% represents an independent risk factor for complications. Thus, patients with a great reduction in serum albumin may benefit from more intense postoperative observation. It may also be helpful in managing the appropriate time for discharge, especially in patients following the ERAS protocol.

In contrast to previous findings [8–10] postoperative albumin alone did not correlate with complications in our study. In line with that, substitution of albumin has not been shown to reduce postoperative morbidity [30]. This further supports the findings that reduction of albumin postoperatively is a marker for surgical stress and albumin per se is not responsible for worse outcome.

A remarkable finding was that patients with an inflammatory mass found at the time of surgery had a significantly better postoperative outcome. This is in contrast to what one would assume. This finding may indicate that different disease phenotypes of CD tend to have different surgical outcome. Even though we could not find any correlation between other disease characteristics such as stenosis or fistula.

There are some limitations that need to be addressed. Although we included a large number of patients, the study data were collected retrospectively. Therefore, we are confronted with the limitations of a retrospective study most importantly selection bias due to missing data.

## Conclusion

Δ albumin is a promising and significant prognostic marker for an eventful postoperative course after laparoscopic surgery for symptomatic CD. Its predictive role was found to be superior to albumin levels alone and at a cut-off of 24.27% Δ albumin is independently associated with postoperative complications.

## Supporting information

S1 Table Data set. Raw data with anonymized basic information.
(XLSX)

## Abbreviations

BMI: Body mass index
ERAS: Enhanced Recovery After Surgery
CD: Crohn’s disease
IBD: inflammatory bowel disease
AUC: area under the curve
ROC: receiver operative characteristic
PEM: protein-energy malnutrition
CI: confidence interval
SPSS: Statistical Package for the Social Sciences
